# Common eye diseases in school going children

**Published:** 2017

**Authors:** P Vijayalakshmi, Sathya T Ravilla

**Affiliations:** Chief Paediatric Opthalmologist, Aravind Eye Hospital; Assistant Professor, Aravind Eye Hospital

**Figure:**
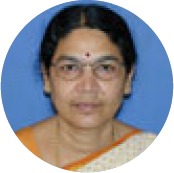
Dr P Vijayalakshmi

**Figure:**
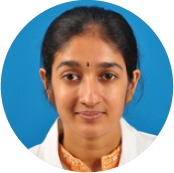
Dr Sathya T Ravilla

**In this article we present an overview of common eye diseases or ailments identified in school-aged children in South Asia with different ways to screen and diagnose them at schools.**

**Figure:**
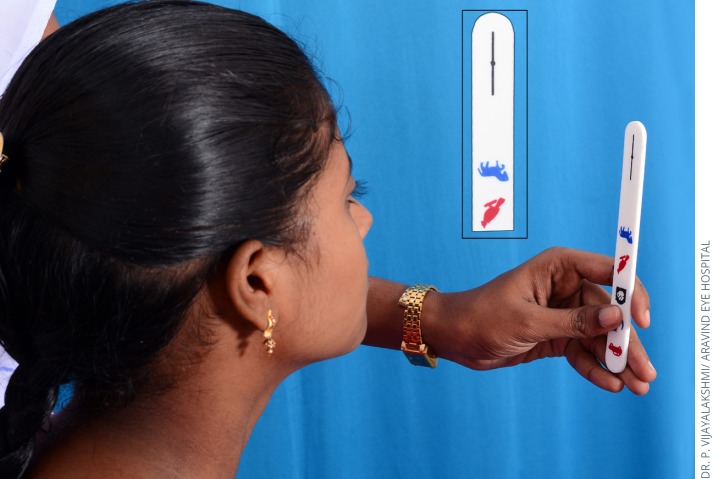
Examination of convergence. INDIA

A part from refractive errors, the other ocular problems which may be encountered in this age group are:

- Strabismus,
- Amblyopia,
- Developmental cataracts,
- Inherited retinal dystrophies,
- Nystagmus,
- Ocular allergies,
- Vitamin A deficiencies and,
- Trauma-related problems and low vision.

## Headache

This is a frequent yet insignificant complaint in this population. However, all children with headache need to be thoroughly evaluated to rule out optic disc edema, refractive error and fusion or accommodative imbalance. Headache and asthenopia due to manifest hypermetropia (MH), insufficient accommodation or poor convergence and fusion can negatively influence a child's academic performance. They may present with blurred vision while reading for near vision tests and face difficulty in changing the focus from near to distance. Manifest hypermetropia is said to be present when a child with unaided visual acuity of 6/6 maintains the same vision even with an addition of +1.5 DS in front of both eyes. Convergence is the mechanism wherein both eyes move towards the nose to focus on near activities. Convergence weaknesses is said to be present when the near point of convergence recedes beyond the normal level of 10 to 12 cm.

Screening for MH can be done by making all children with 6/6 acuity without correction to read the VA chart with +1.5 DS spectacles. Those who can still read 6/6 with the +1.5 DS add need to be referred for retinoscopy and further examination. The amount of convergence can be checked by using a small stick (see picture) printed with a vertical line intercepted by a central dot. The child is asked to focus on the central dot and to notice if the single line tends to appear double, as the examiner slowly brings the stick from 40 cm to 10 cm towards the nose of the child. During the process, the examiner encourages the child to concentrate on the dot, and the examiner looks for any ocular deviation. If the child sees the line as double and/or if a deviation is noticed when the stick is at a distance beyond 10–12 cm, the child needs to be referred to the base hospital for further evaluation. MH is corrected with spectacles, whereas convergence and fusion weakness can be improved with appropriate orthoptic exercises.

## Strabismus and Amblyopia

With two eyes set in the straight ahead position, there is an advantage of having a wider field of vision and a capacity of perceiving the depths of different objects (stereopsis or 3D vision). Strabismus is said to be present when one eye deviates from the straight ahead position (see picture showing left esotropia and right esotropia on page 8). It can occur in one eye or alternately in both eyes. Strabismus may be hereditary, but can also occur due to uncorrected refractive errors, paralysis of the nerves involved with ocular movement or due to obstruction of vision in one eye by ptosis, corneal opacity, cataract, etc. If not detected and treated early, it might result in irreversible visual loss in the deviating eye (amblyopia or lazy eye). *Detection and management:* The deviation of eyes from primary position can easily be detected by shining a torch light at a distance of 40 cm in front of both eyes and noting the position of light reflex on the cornea. Normally they are positioned at the centre of the cornea in both eyes. Depending upon the direction and amount of ocular deviation, the corneal light reflex in that eye gets displaced. Strabismus may be corrected with spectacles in the presence of refractive errors.

Occlusion therapy and reading exercises are given for any associated amblyopia. Surgical alignment of the eyes may be indicated for improving vision or to restore normal alignment of the eyes.

## Cataract

Developmental or traumatic cataract is usually detected by a torch light where the pupil either appears grey or white, with associated decreased visual acuity. Treatment by surgical removal of the cataractous lens followed by implantation of a suitable intra-ocular lens (IOL) may be indicated. It is important to mention that these children will need spectacles post-operatively especially for near vision and long term follow-up with frequent replacement of spectacles.

**Figure:**
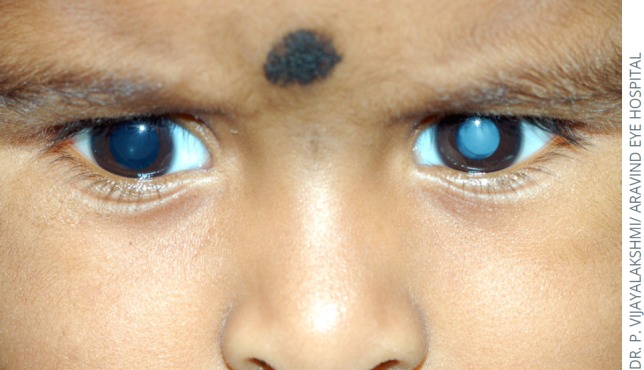
Bilateral cataract with a grey pupil on right side and white pupil on the left side. INDIA

## Nystagmus and low vision

Involuntary or unsteady movements of the eye balls is called nystagmus. Children with nystagmus tend to keep their eyes, face or head turned towards the direction of least eye movements. Nystagmus occurs due to pathology in the eye structures (globe anomalies, retinal dystrophies, etc.) or in the eye movement control system. It is usually associated with poor visual acuity and/or difficulty in reading. A torch light can be used to detect any abnormal eye movements. Initially, refractive errors need to be corrected. Where the vision cannot be improved, support to use residual vision to suit their needs by guidance at a visual rehabilitation unit in a secondary/tertiary eye care centre is recommended.

## Ocular allergies

Vernal kerato-conjunctivitis or seasonal allergic conjunctivitis can cause great discomfort affecting academic performance. It is characterized by severe itching, discharge and foreign body sensation. It can sometimes lead to refractive errors, mostly astigmatism. Since the course of the disease is long, one should be educated about preventive measures and safe use of drugs and avoiding self-medication, particularly steroids.

## Vitamin A deficiency

Presence of Bitot's spots in this population gains importance only when it is associated with night blindness and other associated nutritional issues. Where necessary, this can be managed with a Vitamin A oral supplementation, as recommended by WHO.

**Figure:**
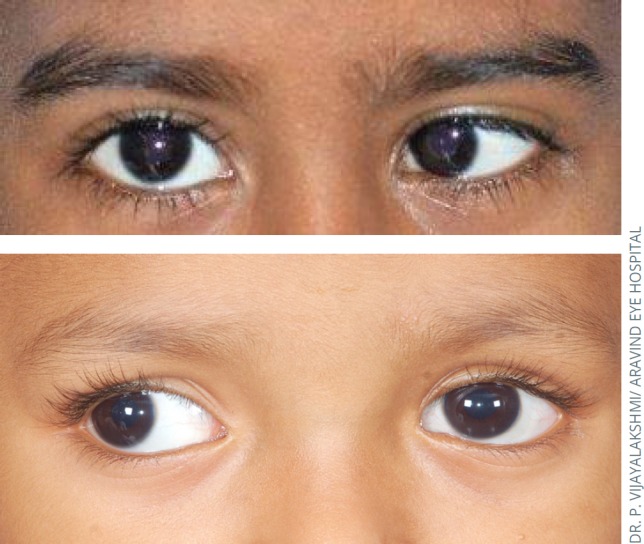
Top: Left esotropia showing the deviation of the left eye inward along with displacement of the light reflex in the cornea, temporally. Bottom: Right exotropia displaying the deviation of right eye outward along with displacement of the light reflex in the cornea, nasally. INDIA

A vision screening process for detecting uncorrected refractive error should include the following examinations to make it a comprehensive examination:

- Use +1.5 DS test to detect manifest hypermetropia
- During a headache with eye strain, use a target stick to look for convergence insufficiency
- Examination with a torch light, to look for strabismus, nystagmus, white reflex at the pupil (cataract), small or large eye balls, and any other abnormalities in the lids,
- Examination of the conjunctiva to look for bitot's spots or signs of allergy
- Explain any abnormal findings to the children/parents/teachers and encourage them to undergo further examinations at a designated place.

## Challenges

The above examination requires a slightly longer time and some training of the fieldworker. Investing in training of the technician makes the programme more comprehensive. Apart from having sufficient funding, processes need to be in place to keep track of children who need secondary and tertiary care to ensure complete care.

**Figure:**
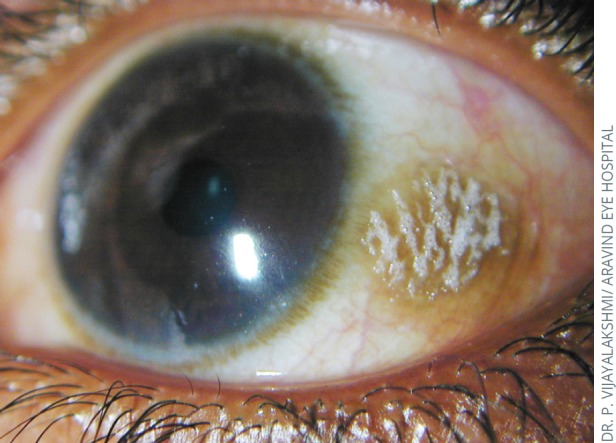
Bitot's spot on the temporal conjunctiva. INDIA

